# Evaluation of Human Serum Albumin Nanoparticles for Rapamycin Delivery

**DOI:** 10.1021/acsomega.6c02977

**Published:** 2026-08-04

**Authors:** Camila Fernanda Rodero, Cristina Pangua, Jorge Morales Gracia, Melibea Berzosa Suner, Marcela Tavares Guiguer, Marlus Chorilli, Juan M. Irache

**Affiliations:** † Department of Drugs and Medicines, School of Pharmaceutical Sciences, São Paulo State University, Araraquara 14800-903, Brazil; ‡ Department of Pharmaceutical Sciences, 16754 University of Navarra, Pamplona 31008, Spain; § Department of Microbiology and Parasitology, University of Navarra, Pamplona 31008, Spain

## Abstract

Rapamycin (RAP) is a macrolide antibiotic with potent immunomodulatory and anticancer properties, but its clinical use is hindered by poor solubility, chemical instability, and limited bioavailability. The aim of this study was the development of PEG-coated albumin nanoparticles for delivering rapamycin and maintaining its biological activity after encapsulation. Rapamycin-loaded albumin nanoparticles were prepared by desolvation of an aqueous solution of the protein with ethanol and subsequent coating with PEG 35,000. The resulting nanoparticles (NPA-RAP) displayed adequate physicochemical properties for intravenous delivery, including sizes within the tumor-targeting range (150–200 nm), low polydispersity (<0.2), and negative zeta potential (∼−30 mV). PEGylation and surface charge ensured short-term stability, while encapsulation efficiency exceeded 70%, with sustained biphasic release (40% over 24 h) well described by the Weibull model. In vitro, NPA-RAP showed cytotoxicity in MCF-7 breast cancer cells comparable to free rapamycin but with lower IC_50_ values. In *Caenorhabditis elegans*, NPA-RAP did not affect fat content but extended lifespan by ∼70%, outperforming free rapamycin. These results suggest that these nanoparticles can successfully transport rapamycin without causing detectable carrier-induced toxicity, highlighting its promise for further therapeutic assessment.

## Graphical abstract

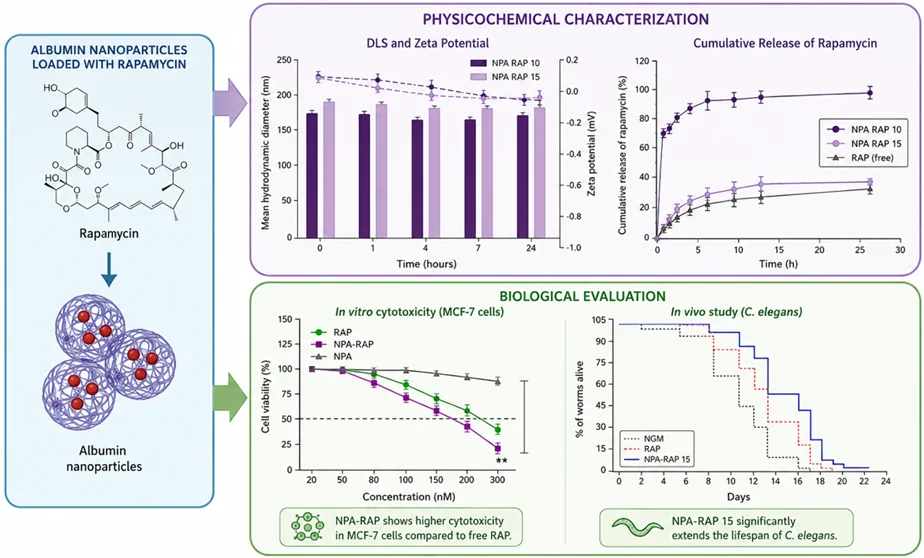

## 1 Introduction

Rapamycin (RAP) is a lipophilic macrolide antibiotic first isolated from *Streptomyces hygroscopicus* during the mid-1970s.^1^ The mammalian target of rapamycin (mTOR) is a 289 kDa serine/threonine kinase belonging to the phosphatidyl inositol 3-kinase family. mTOR is located in the cytoplasm, where it regulates cell growth and cell cycle progression by responding to nutrients (such as amino acids) and growth factors.^2,3^ Several studies have reported that RAP binds intracellularly to FKBP-12 (FK506-binding protein 12), forming a complex that subsequently interacts with the FKBP12-rapamycin binding (FRB) domain of the mTOR protein. This interaction inhibits growth factor signaling and induces cellular apoptosis.^3^

Through modulation of mTOR signaling, rapamycin exerts both immunosuppressive and immunostimulatory actions within innate and adaptive immune responses.^4^ Its capability to suppress immune activity underlies its clinical success in preventing organ transplant rejection,^5^ whereas its immunostimulatory effects would contribute to its antitumor potential.^6^ These outcomes would be at least partially dependent on the dose and dosing regimen, with lower doses favoring immune activation and intermittent administration promoting immune function while minimizing metabolic and immunosuppressant effects.^7^ Consequently, rapamycin displays diverse biological activities, including antifungal, antiproliferative, and antineoplastic properties.^8,9^ Within this framework, RAP has been explored as a potential therapeutic candidate for breast cancer, the second most prevalent cancer globally and the leading malignancy in women, representing roughly 30% of female cancer cases.^9^

Despite its therapeutic potential, the clinical application of RAP is limited by its very poor solubility, both in water (2.6 μg/mL) and in the conventional excipients used in injectable formulations,^10^ and chemical stability during manufacturing and storage (sensitive to light, temperature, and pH changes).^11^ In addition, after injection, the availability of free rapamycin is limited due to a strong tendency to be sequestered in erythrocytes.^12^

To solve these drawbacks and improve the effect of rapamycin, various alternatives based on the use of nanostructured devices have been proposed, including polymer nanoparticles,^13^ liposomes,^14^ micelles,^15^ nanoemulsions,^11^ dendrimers,^16^ and nanogels.^73^ Overall, this strategy allows one to increase the amount of drug introduced in the organism, prolong the circulation time in the bloodstream, reduce potential drug-related side effects, and improve patient compliance by facilitating more effective and better-tolerated treatments.^17,18^

Another alternative for the formulation of rapamycin could be its encapsulation in human serum albumin nanoparticles. In addition to their biodegradability and regulatory status, albumin-based nanoparticles offer important advantages, including a relatively prolonged circulation time and an intrinsic ability to interact with tumor-associated receptors such as gp60, SPARC, and FcRn.^19,20^ Owing to these benefits, albumin-based nanoparticles have been commercialized (Albunex, Abraxane, and Fyarro) and are currently used in clinical therapy.^19,21^ In this context, the aim of this study was to conduct a preliminary evaluation of the potential of albumin nanoparticles for delivering rapamycin and maintaining its biological activity after encapsulation. For this purpose, PEG-coated albumin nanoparticles were assessed in vitro in breast cancer cells and in vivo in a *Caenorhabditis elegans* model.

## 2 Materials and Methods

### 2.1 Materials

Human serum albumin lyophilized power (HSA), polyethylene glycol 35,000 (PEG 35,000), sucrose, rapamycin, Nile red, Orlistat, dimethyl sulfoxide (DMSO), 5-fluoro-2′-deoxyuridine (FUdR, 98%), and Trypsin–EDTA were purchased from Sigma-Aldrich (St Louis, MO). Gibco phosphate-buffered saline (PBS) was purchased from Thermo Fisher Scientific (Waltham, MA). Taxol was donated by the hospital of clinics in Ribeirão Preto/São Paulo. Endothelial Cell Growth Basal Medium (MEBM) and Dulbecco’s modified Eagle’s medium (DMEM) were purchased from Lonza and Gibco, respectively. Bovine Fetal Serum (FBS) and 1% antibiotic/antimycotic solution (10,000 units penicillin, 10 mg streptomycin, and 25 μg amphotericin B per mL) (Sigma-Aldrich). Resazurin solution (Sigma-Aldrich). Lumogen F Red was donated by BASF (Ludwigshafen, Alemanha).

### 2.2 Synthesis of Human Serum Albumin Nanoparticles

Nanoparticles were prepared by the desolvation method, prior stabilization by incubation with PEG and, finally, freeze-dried.^22^ Briefly, 100 mg HSA were dissolved in 7.5 mL of water, the pH was adjusted to 4.9 with hydrochloric acid 1 M. Nanoparticles were obtained through the continuous dripping of 16 mL ethanol containing either 10 or 15 mg rapamycin under constant magnetic stirring. Then, albumin nanoparticles were incubated with 500 μL of PEG 35,000 solution (10%, v/v) 30 min under magnetic agitation. The solvent was removed under reduced pressure using a rotary evaporator (Büchi Rotavapor R-144; Büchi, Postfach, Switzerland) and the nanoparticles were purified and concentrated by ultrafiltration through a polysulfone membrane cartridge (50 kDa) (Medica SPA, Italy). Finally, the formulations were frozen and lyophilized (Genesis 12EL, Virtis, NewYork, USA) in the presence of 5% sucrose as a cryoprotectant agent. The nanoparticles prepared with 10 and 15 mg of RAP were named as NPA-RAP10 and NPA-RAP15, respectively. Empty nanoparticles (NPA) were produced with the absence of rapamycin and employed as control. For in vivo studies, Lumogen Red-loaded albumin nanoparticles were prepared by dissolving 5 mg of Lumogen Red in the hydroalcoholic solution during the formulation process.

### 2.3 Physicochemical Characterization of Human Serum Albumin Nanoparticles

#### 2.3.1 Size, Polydispersity Index, and Zeta Potential

Nanoparticle size distribution and polydispersity were characterized by photon correlation spectroscopy, whereas zeta potential was assessed by electrophoretic laser Doppler anemometry. All measurements were performed using a Zeta Plus analyzer apparatus (Brookhaven Inst. Corp., USA) at 25 °C and after dispersion of nanoparticles in ultrapurified water.

#### 2.3.2 Stability of Nanoparticles

The nanoparticles’ stability was evaluated at 25 °C during 24 h after dispersion in PBS (pH 7.4). At predetermined times; the size, polydispersity index, and zeta potential of nanoparticles were measured.

#### 2.3.3 Quantification of RAP by High-Performance Liquid Chromatography

The amount of rapamycin loaded in albumin nanoparticles was calculated by HPLC-UV following a previously reported method^13^ with modifications. Chromatographic analyses were performed in an Agilent 1100 series liquid chromatography system equipped with diode array detector set at 277 nm. The HPLC system was carried out with a Zorbax Eclipse XDB-C18 column (150 mm × 4.6 mm, 5 μm; Agilent, Torrance, USA). The mobile phase methanol/water (76:24, v/v) was pumped at 0.5 mL/min. The column temperature was maintained at 45 °C, and 10 μL sample was injected for each analysis. The amount of RAP encapsulated in albumin nanoparticles was determined using the centrifugation method (*n* = 3). For this purpose, formulations were centrifuged at 3500*g* for 20 min. The pellets containing the unloaded RAP were dissolved in methanol and analyzed by HPLC to determine the amount loaded in nanoparticles. The encapsulation efficiency (EE) was assessed as the quotient between the total amount of rapamycin recovered in the pellet after centrifugation and the total amount of rapamycin added in the nanoparticles.

#### 2.3.4 In Vitro Release of Rapamycin in Human Serum Albumin Nanoparticles

The release profile of rapamycin from albumin nanoparticles was carried out at pH 7.4 and 37 ± 0.5 °C, under sink conditions, in individual Slide-A-Lyzer G2 Dialysis Cassettes devices (MWCO 20,000) (Thermo Fisher Scientific, Rockford, USA). The cassettes, filled with 17.2 mg rapamycin-loaded nanoparticles dispersed in 0.5 mL water, were added into a vessel containing 80 mL PBS (pH 7.4) and 1% sodium lauryl sulfate, under stirring at 150 rpm. At predetermined intervals, samples were collected, filtered using syringe filters (0.45 μm) (Thermo Scientific), and RAP quantified amount by HPLC, determining which mathematical models best apply to this formulation.

To investigate the drug release mechanism, the experimental data were fitted to four mathematical models: Korsmeyer–Peppas,^23^ Weibull,^24,25^ Higuchi,^26^ and First-order.^27^

The Korsmeyer–Peppas model describes drug release when the mechanism is complex, involving a combination of Fickian diffusion and non-Fickian (anomalous) transport. The model is expressed by eq 1

1 $$Q_{t}=At^{n}$$

where *n* indicates the type of release mechanism.

The Weibull model (eq 2) describes drug release over time using three parameters (*k*, *t*_c_, *d*), allowing the adjustment of different curve shapes (exponential, sigmoidal, or parabolic).

2 $$\frac{M_{t}}{M_{\infty }}=1-e^{-{\left(k\left(t-t_{c}\right)\right)}^{d}}$$

*k* is 1/*a*, where “*a*” is the time–scale parameter, which is determined empirically; *t* represents the time elapsed since administration; *t*_c_ is the location parameter, representing the lag time before the onset of release; *d* is the shape parameter, which determines the curve type.

The Higuchi model (eq 3) is applicable to drug release from matrix systems where diffusion is the primary mechanism, particularly when the drug is uniformly distributed within the matrix.^26^ The equation assumes release is controlled by both swelling and erosion processes.

3 $$Q_{t}=k_{H}t^{1/2}$$

where *Q*_*t*_ is the cumulative amount of drug released at time *t*, whereas *k*_H_ is the Higuchi dissolution constant.

Finally, the First-order kinetics model (eq 4) describes drug release as being proportional to the remaining drug concentration. It reflects a decreasing release rate over time

4 $$Q_{t}=Q_{\infty }\left(1-e^{-k_{1}t}\right)$$

where *Q*_∞_ is the total amount of drug released, *Q*_*t*_ is the amount released at time *t*, and *k*_1_ is the first-order release constant.

### 2.4 Biological Assays

#### 2.4.1 In Vitro Cytotoxicity Assessment of Human Serum Albumin Nanoparticles

In vitro cytotoxicity studies of albumin nanoparticles were performed on MCF-7 and MCF-10A cell lines, following previously described protocols.^28,29^ MCF-7 cells were cultured in DMEM and MCF-10A cells in MEGM, both supplemented with FBS (10%, v/v) and antibiotic/antimycotic solution (1%, v/v). Cells were cultured at 37 °C in a humidified incubator containing 5% CO_2_. Upon reaching approximately 90% confluence, cells were harvested, resuspended, and seeded into 96-well plates at a density of 2.5 × 10^5^ cells/mL. After 24 h of incubation, wells were washed with PBS (pH 7.4), and the experimental treatments (free rapamycin, NPA, NPA-RAP10, and NPA-RAP15) diluted in culture medium were applied. Cells were incubated for a further 24 h. Cell viability was subsequently determined using the resazurin reduction assay. This method relies on the conversion of resazurin into the pink fluorescent metabolite resorufin by viable, metabolically active cells. For this purpose, wells were washed with PBS and a resazurin solution was added. After 4 h, fluorescence was determined at 540 nm excitation and 590 nm emission using a Thermo Multiskan EX microplate reader. The half-maximal inhibitory concentration (IC_50_) was calculated from the resulting cell viability curves. All experiments were conducted in triplicate.

#### 2.4.2 In Vivo Evaluation in *C. elegans*

##### 2.4.2.1 Strains and Cultivation

The wild-type N2 Bristol strain of *C. elegans* and *Escherichia coli* OP50 bacterial stain were supplied by the Caenorhabditis Genetics Center (CGC, University of Minnesota). The nematode diet consisted of *E. coli* OP50. Maintenance of all strains was performed at 20 °C on Nematode Growth Medium (NGM) agar plates.^30^

##### 2.4.2.2 Treatment with Rapamycin-Loaded NPA

The experiments were carried out in triplicates in 6-well plates containing 4 mL NGM per well supplemented with glucose (50 mM). Orlistat (6 μg/mL) was employed as the positive control to evaluate fat reduction.^31^ The effect of rapamycin dissolved in DMSO 1% was tested at different concentrations (37.5–150 μM). The effect of NPA-RAP10 and NPA-RAP15 (after their dispersion in water) was investigated at a drug concentration of 75 μM. After the addition of treatments to the wells containing NGMs, they were mixed and left to solidify and dry in dark conditions. The plates were solidified and, after 2 days, 100 μL of *E. coli* OP50 suspension was added to each well. For each experiment, age synchronized population were obtained by treating the worms with sodium hypochlorite treatment, followed by incubation of the eggs in M9 buffer for 18 h. The resulting larvae in L1 stage (around 300 to 500 individuals) were transferred onto the plates before incubation for 46 h at 20 °C until the larvae reached the L4 stage.^32^

##### 2.4.2.3 Nanoparticles Intake

The nanoparticles ingestion by *C. elegans* was evaluated by incubating age-synchronized worms on NGMplates with nanoparticles fluorescently labeled with Lumogen F Red. Following a 2 h of exposure period, the worms were washed three times with M9 medium, with each wash lasting 30 s. Then, they were placed onto slides prepared with fresh 2% agarose pads containing sodium azide (1%). The presence of nanoparticles in the worms’ intestines was evaluated through the measurement of nanoparticles fluorescence using a fluorescence microscope (Axio imager M1 Zeiss, Jena, Germany) equipped with a Axiocam Icc3 digital camera and an HBO 100 fluorescent light source. Zen software (Zeiss) was used to obtain the images.^33^

##### 2.4.2.4 Fat Content Quantification

The fat content in the worms was quantified through the fixative-based Nile Red method.^33^ In brief, L4-stage worms maintained under the different experimental conditions were harvested and washed twice with 1 mL PBS containing 0.01% Triton-X (PBST). Then, the worms were left for 15 min on ice and fixed in 40% isopropanol. Nile Red solution (3 μg/mL) was added and the plates were incubated for 30 min in dark conditions. After a final wash with PBST, the worms were mounted on 2% agarose slides and examined under a microscope. Fluorescent images were acquired at 80× magnification using a Nikon SMZ18 stereomicroscope (Nikon Instruments Inc., Tokyo, Japan) equipped with an epifluorescence illumination system and a DS-FI1C refrigerated color digital camera (Nikon). Image acquisition was performed with GFP filter set (Ex 480–500, DM 505; BA 535–550), and the resulting images were analyzed using ImageJ software.

##### 2.4.2.5 Lifespan Analysis

The effect of the nanoparticles in the longevity of *C. elegans* was investigated at 20 °C under high glucose conditions.^34^ To prevent progeny production, worms were maintained on NGM plates and supplemented with 40 mM FUdR to prevent the growth of new worms. The prefertile period adult stage was designated as time zero (*t* = 0). Live worms were counted daily, and dead worms were removed from the plates after each assessment. Nematodes were considered dead when they failed to respond to repeated mechanical stimulation. All experiments were repeated on three different days and in triplicate on each day, each experiment was performed using approximately 50 worms.

### 2.5 Statistical Analysis

Data were presented as the mean ± standard deviation (SD) from at least three independent experiments. Statistical comparisons among groups were analyzed by one-way ANOVA followed by Tukey’s post hoc test. Statistical significance was defined as *p* < 0,01. All analyses were conducted using GraphPad Prism 6.0 (La Jolla, CA).

## 3 Results and Discussion

### 3.1 Physico-Chemical Characterization of Rapamycin-Loaded Albumin Nanoparticles

The physicochemical characteristics of nanoparticles (particularly their size and surface properties) play a decisive role in their biodistribution within the body, and consequently, in their potential efficacy and/or toxicity. Among these attributes, particle size is especially critical, as it strongly influences the biological activity and therapeutic effect of nanoparticles, including their activity against breast cancer. In general, nanoparticles smaller than 200 nm demonstrate an enhanced capacity to permeate the microvasculature and accumulate in the tumor microenvironment via passive targeting mechanisms.^35^ Reduced particle size also enhances deeper penetration into tumor tissue, which is essential for improving therapeutic outcomes. However, particles smaller than 5 nm are rapidly eliminated from circulation through glomerular filtration and renal excretion.^36,37^ Consequently, the optimal size range for nanoparticles in intravenous drug delivery is generally 10–200 nm,^38^ allowing sufficient circulation time, tumor accumulation, and avoidance of rapid clearance by the body’s filtration systems.^39^

In our study, unloaded albumin nanoparticles (NPA) exhibited a mean particle size below 150 nm (Table 1), which falls within the optimal range for tumor accumulation and tissue penetration.^40^ Incorporation of RAP into the formulations (NPA-RAP10 and NPA-RAP15) increased the mean particle size to approximately 200 nm (Table 1). Furthermore, increasing the RAP-to-albumin ratio produced a proportional increase in the nanoparticle size (Table 1).

**Table 1:** Physicochemical Characteristics of Rapamycin-Loaded Nanoparticles Prepared at a Drug-to-Albumin Ratio of Either 0.1 (NPA-RAP10) or 0.15 (NPA-RAP15)^a^

	size (nm)	PDI	zeta potential (mV)	rapamycin loading (μg/mg)	EE (%)
NPA	143 ± 8	0.180 ± 0.062	–30.2 ± 6.5	-	-
NPA-RAP10	181 ± 3	0.063 ± 0.027	–30.1 ± 3.2	26.27 ± 0.22	71.7 ± 0.16
NPA-RAP15	200 ± 4	0.115 ± 0.055	–34.4 ± 5.4	46.30 ± 0.12	80.9 ± 0.11

a Data expressed as mean ± SD (*n* = 3). PDI: polydispersity index; EE: encapsulation efficiency.

Besides the mean particle size, the polydispersity index (PDI) is an important parameter for assessing the homogeneity of nanoparticle populations, which impacts their biological performance. All formulations exhibited a PDI below 0.2, indicating a narrow size distribution and high uniformity.^41^ These findings are consistent with the desolvation method used for nanoparticle preparation (2016) and also reported albumin nanoparticles produced via desolvation with particle sizes around 100 nm and PDIs below 0.2, supporting the robustness of this method.^42^

The zeta potential of all nanoparticle formulations was approximately −30 mV. The incorporation of RAP did not significantly alter the surface charge. Similar results have been reported by Galiyeva et al. (2023), who observed stable albumin nanoparticles with comparable zeta potential values and an EE of 44% for rifampicin, further supporting the system’s stability and potential for drug delivery applications. Nanocarriers with negative surface charges present significant advantages for intravenous administration, primarily due to the electrostatic repulsion that reduces nanoparticle aggregation and enhances colloidal stability in biological fluids.^43^ Beyond this, PEGylation of albumin nanoparticles further prolongs blood circulation time by providing a hydrophilic steric barrier that diminishes recognition and clearance by the reticuloendothelial system, improving systemic bioavailability.^44^

Importantly, both the negative surface charge and PEGylation contribute not only to enhanced in vivo performance but also to maintaining nanoparticle stability during storage. Zeta potentials with absolute values of approximately ±30 mV or higher generate sufficient electrostatic repulsion to prevent particle aggregation over time.^45^ Meanwhile, PEG chains create steric hindrance that acts synergistically with electrostatic forces to stabilize nanoparticles through both steric and electrostatic mechanisms.^46^

In our study, the 24 h stability of the developed albumin-based nanoformulations was systematically evaluated (Figure 1) to confirm that the combined effects of surface charge and PEGylation effectively prevented aggregation, ensuring formulation robustness essential for clinical translation.

**Figure 1:**
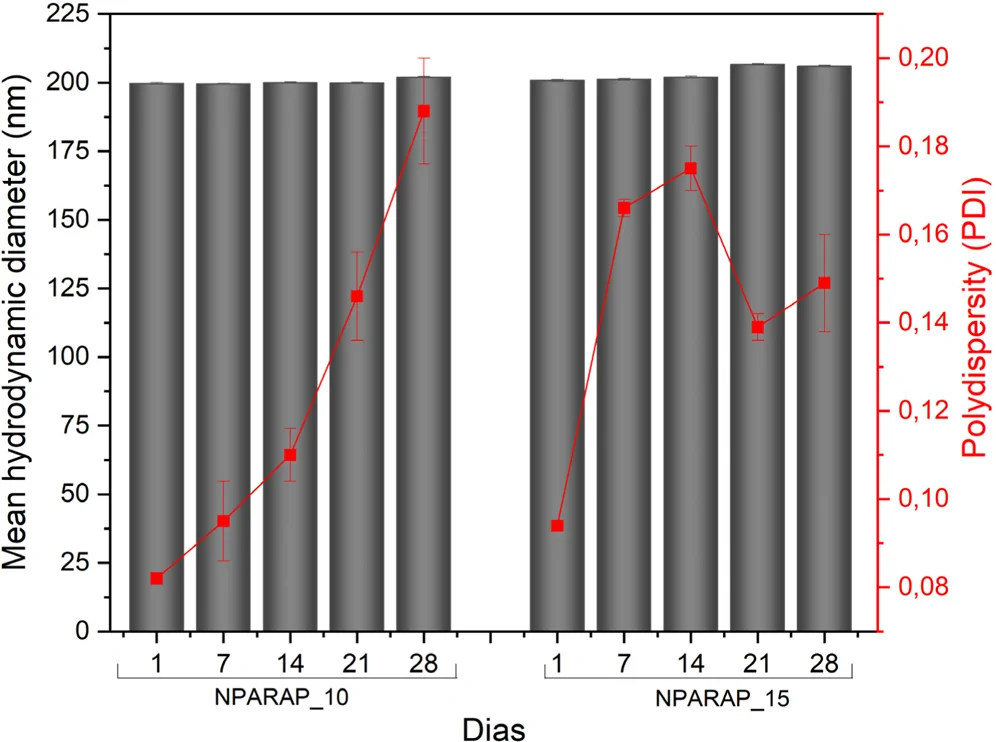
Results of the average size and polydispersity index of human serum albumin nanoparticles (NPA-RAP10 and NPA-RAP15) after their dispersion in PBS. Data expressed as mean ± SD, *n* = 6.

The EE of RAP in albumin nanoparticles was evaluated at two different drug-to-albumin ratios (0.1 and 0.15). In all cases, the resulting formulations (NPA-RAP10 and NPA-RAP15) exhibited high EE (>70%), with NPA-RAP15 achieving the highest value (about 81%; Table 1). Increasing the drug-to-albumin ratio enhanced RAP loading, which rose from 26.27 μg RAP/mg nanoparticles (NPA-RAP10) to 46.30 μg RAP/mg nanoparticles (NPA-RAP15). Similar encapsulation capabilities of albumin nanoparticles for hydrophobic compounds have been reported previously, including paclitaxel,^47^ docetaxel,^48^ or curcumin.^49^

Following comprehensive physicochemical characterization, the in vitro release profile of RAP was evaluated (Figure 2). Drug release from nanosystems can be influenced by various factors, including the physicochemical properties of RAP and the nanoparticle composition.^50^ RAP-loaded nanoparticles exhibited a biphasic release pattern, characterized by an initial burst of approximately 20% of the loaded drug within the first 2 h, followed by a slower and more sustained release phase extending up to 24 h, reaching around 40% of the total payload. Notably, the RAP payload did not significantly affect the release profiles. By contrast, under the same experimental conditions, nearly 90% of free RAP diffused across the membrane within the first 2 h.

**Figure 2:**
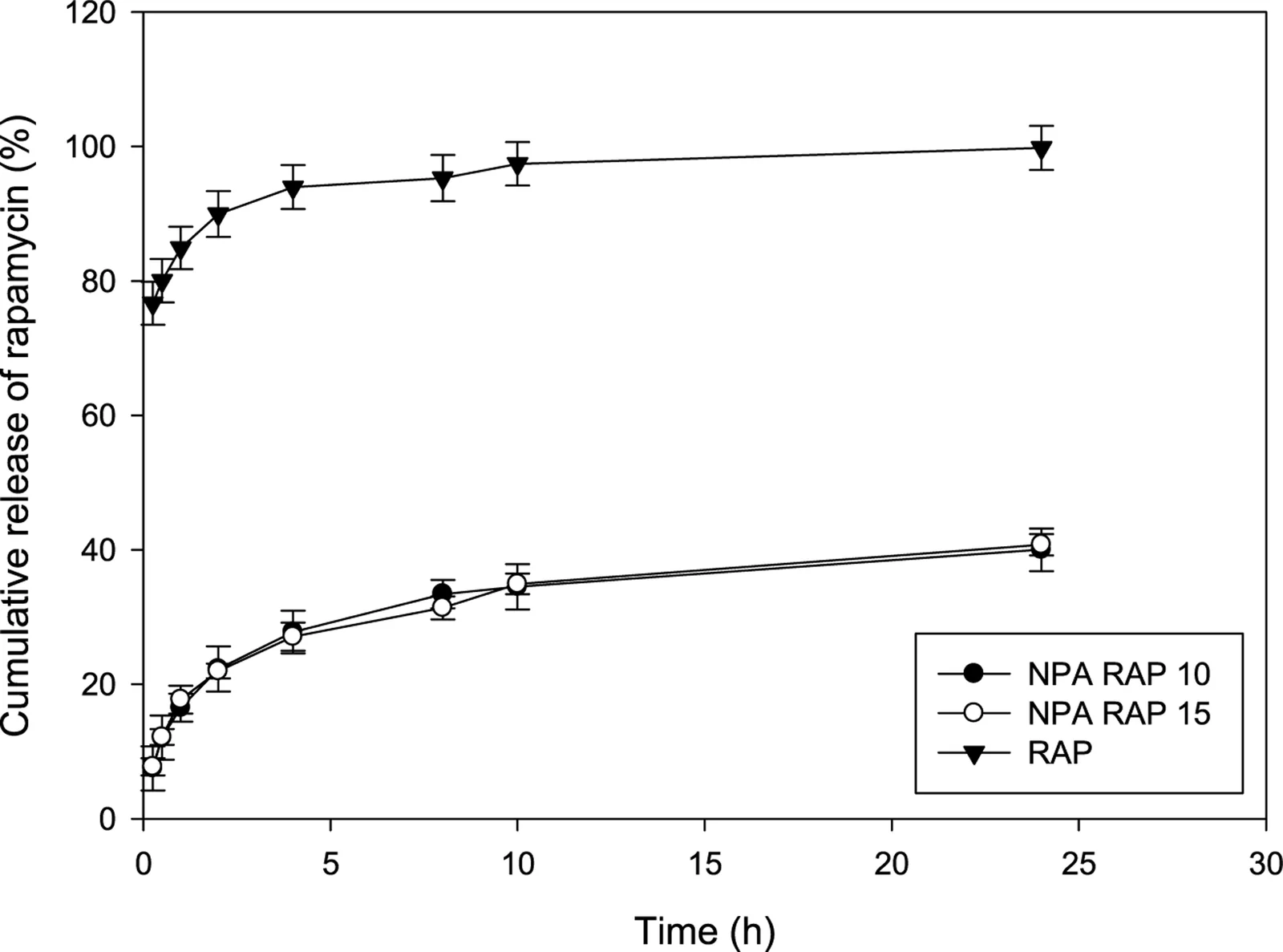
Rapamycin release profile from albumin nanoparticles (NPA-RAP 10 and NPA-RAP-15) after incubation in PBS (pH 7.4). Data expressed as mean ± SD, *n* = 3.

Mathematical modeling plays a crucial role in analyzing drug release kinetics from nanoparticle-based systems while maintaining a feasible level of complexity. Among the models tested, the Weibull model provided the best fit to the experimental data, with adjusted *R* ^2^ values of 0.999 for NPA-RAP10 and 0.998 for NPA-RAP15 (Table 2). Originally introduced by Weibull (1951), this model is widely employed in pharmaceutical sciences due to its flexibility.^24^ The shape parameter (β) determines the curvature of the release profile, while the location parameter (td) indicates the delay before the onset of drug release. These characteristics enable the Weibull model to accommodate a broad spectrum of release behaviors. Moreover, incorporating a natural logarithmic transformation into the model equation can enhance its adaptability by reducing data fluctuations.^51^ However, it is important to note that the model does not fully capture phase-specific variations in release rates.^51^ Subsequent studies have corroborated both the strengths and limitations of the Weibull model in describing drug release across diverse formulations.^52^

**Table 2:** Release Kinetic Parameters Obtained by Fitting the Drug Release Data of the Formulations to Different Kinetic Models

mathematical models	RAP	NPA-RAP10	NPA-RAP15
	*R* ^2^ = 0.980	*R* ^2^ = 0.978	*R* ^2^ = 0.984
Korsmeyer–Peppas	*K* = 79.85	*K* = 16.88	*K* = 16.81
	*n* = 0.037	*n* = 0.294	*n* = 0.295
Weibull	*R* ^2^ = 0.997	*R* ^2^ = 0.999	*R* ^2^ = 0.998
	*b* = 0.266	*b* = 0.492	*K* = 0.386
Higuchi	*R* ^2^ = 0.700	*R* ^2^ = 0.820	*R* ^2^ = 0.828
	*K* = 31.38	*K* = 10.36	*K* = 10.33
first-order	*R* ^2^ = 0.66	*R* ^2^ = 0.62	*R* ^2^ = 0.650
	*K* = 4.62	*K* = 0.035	*K* = 0.035

Additionally, the nanoparticle morphology and size were further analyzed by transmission electron microscopy (TEM), as shown in Figure 3. For this analysis, nanoparticle suspensions were deposited onto a carbon-coated copper grid and allowed to dry at room temperature. The samples were then observed under TEM to evaluate the particle size, morphology, and structural characteristics.

**Figure 3:**
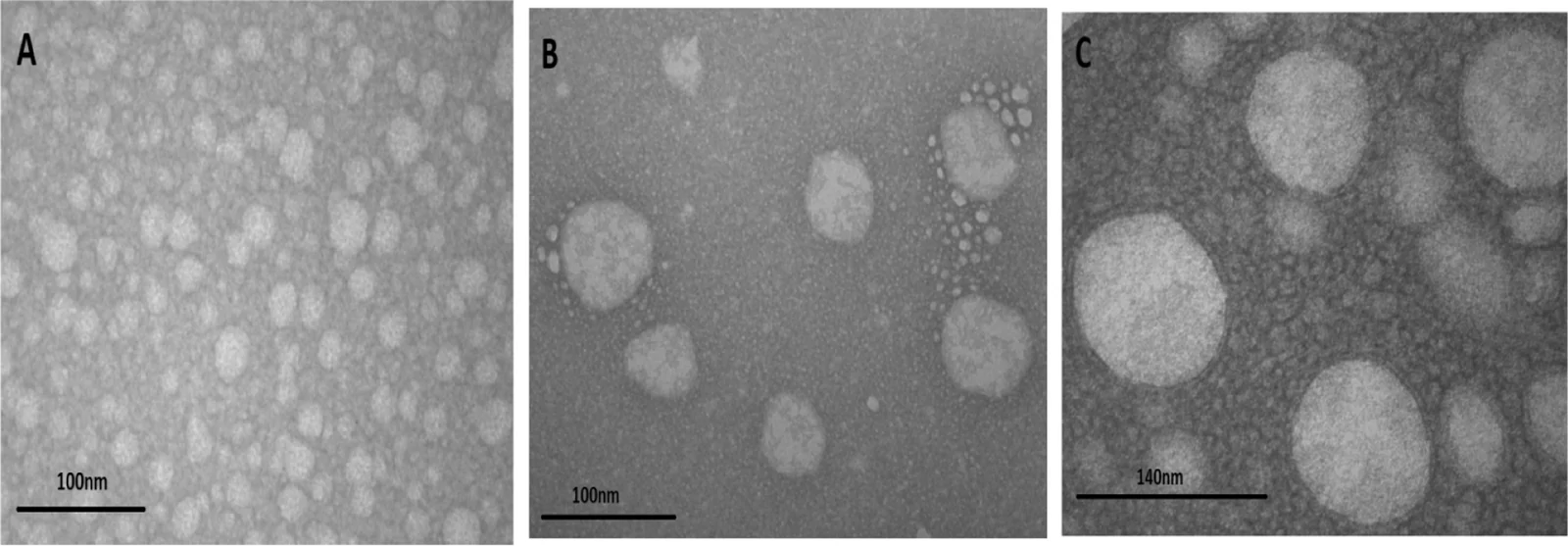
TEM images of nanoparticle formulations. (A) NPA, (B) NP-RAP 10, and (C) NPA-RAP 15. The images show the morphology and size distribution of the nanoparticles, indicating spherical shape and homogeneous structure across the different formulations.

### 3.2 In Vitro Cytotoxicity Assessment of Rapamycin-Loaded NPA Systems

Free rapamycin and NPA-RAP15 were tested at concentrations ranging from 10 to 300 nM in MCF-7 (breast cancer) and MCF-10A (nontumorigenic breast epithelial) cell lines over a 24 h period. The empty nanoparticle formulation (NPA) showed no cytotoxicity (Figure 4), confirming that the observed effects were due to rapamycin and not to the carrier. NPA-RAP15 exhibited a cytotoxicity profile comparable to that of free rapamycin, indicating that the nanoparticle formulation effectively released the drug while potentially protecting it from degradation. The half-maximal inhibitory concentration (IC_50_) values for free rapamycin were 200 nM in MCF-7 cells and 150 nM in MCF-10A cells. Interestingly, treatment with NPA-RAP15 resulted in lower IC_50_ values of 150 nM and 100 nM, respectively. These findings suggest that rapamycin efficiently inhibits cell proliferation^53^ and that encapsulation within albumin nanoparticles may enhance its bioactivity.

**Figure 4:**
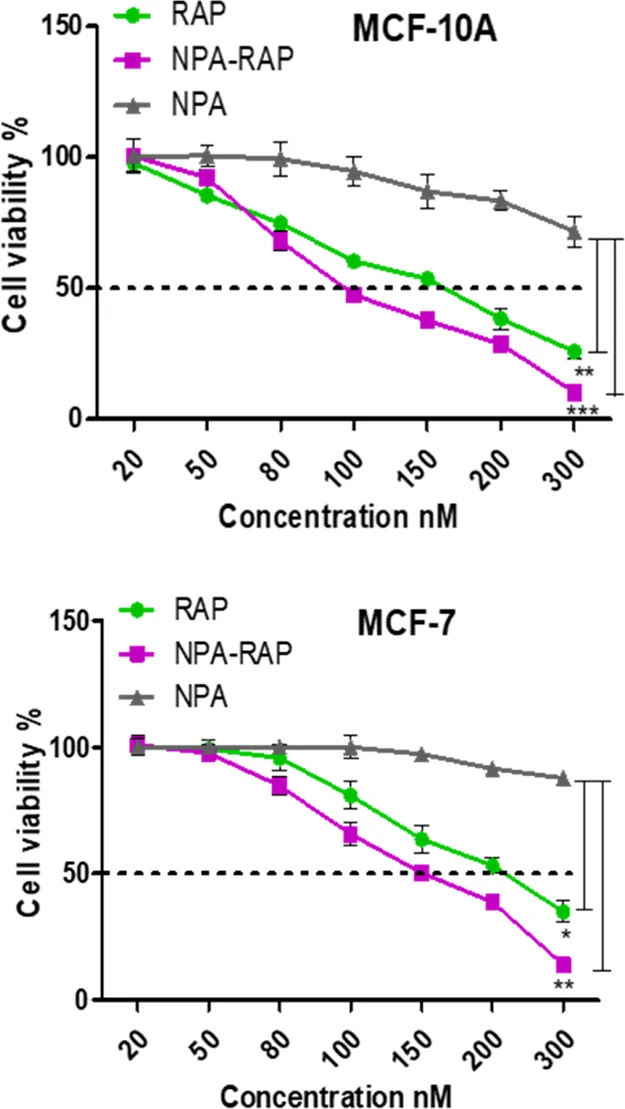
Cell viability of MCF-7 and MCF-10A cells following treatment with empty nanoparticles, free rapamycin, and rapamycin-loaded nanoparticles (NPA-RAP15) at various concentrations (10–300 nM). Experiments were performed in triplicate. Results are presented as mean ± SEM. Statistical analysis was performed using one-way ANOVA. *p < 0.05; **p < 0.01; ***p < 0.001.

Rapamycin’s limited effectiveness during short treatment periods is likely due to its selective inhibition of mTORC1. However, sustained release systems, such as nanoparticle-based delivery, may prolong drug exposure and enable partial inhibition of mTORC2 as well.^54^ Dual inhibition of mTORC1 and mTORC2 has been associated with enhanced therapeutic efficacy, including reduced angiogenesis and decreased cell survival.^55^

### 3.3 In Vivo Study Using *C. elegans*

*C. elegans* is a free-living nematode that inhabits the aqueous microenvironments of soil. It has been widely recognized as a valuable model organism for the biochemical and toxicological evaluation of various xenobiotics, as well as for studies on aging and longevity.^56^ The growing popularity of *C. elegans* in experimental research is largely attributed to its ease of handling, optical transparency, short lifespan, simple cultivation, and rapid generation time. Additionally, the model is highly relevant for biomedical studies, as approximately 60–80% of its genes have human homologues.^57^

#### 3.3.1 Effect of Rapamycin-Loaded Nanoparticles on Fat Content

Rapamycin is a well-characterized inhibitor of the mechanistic target of rapamycin (mTOR), a central regulator of cell growth, metabolism, and aging.^58^ Clinically approved as an immunosuppressant to prevent organ rejection and to treat certain types of cancer, it is also under investigation for its potential in treating neurodegenerative diseases,^12^ cardiovascular disorders,^59^ and diabetes.^60^

The *C. elegans* model is a powerful model organism for studying conserved signaling pathways, including insulin/IGF-1 signaling (IIS) and the mTOR pathway.^61^ The mTOR pathway operates through two functionally distinct complexes: mTORC1, which integrates mitogenic and nutrient signals to regulate cell growth and proliferation, and mTORC2, which modulates cytoskeletal organization and cell morphology.^62^ Both complexes are implicated in key aging-related processes such as lifespan regulation, lipid metabolism, and mRNA translation.^63^

In *C. elegans*, mTOR influences aging through multiple mechanisms, including systemic hormonal regulation. It modulates insulin-like peptides such as *ins-7*, functionally overlapping with *daf-2*, the nematode’s insulin/IGF-1 receptor homologue.^64^ Inhibition of mTORC1 has been shown to increase the expression of daf-16/FOXO target genes related to stress response and detoxification,^65^ as well as upregulate lipl-4, a lipase gene promoting lipolysis.^66^ Interestingly, under reduced mTOR activity, lipl-4 expression may become less dependent on daf-16.^67^ However, the relationship between mTOR inhibition and lipid storage remains complex, as some studies have reported paradoxical lipid droplet accumulation.^68^ Additionally, mTORC1 can act via nontranscriptional pathways, such as phosphorylation of S6 kinase (S6K), a key regulator of protein synthesis and lifespan.^69^

To assess the metabolic effects of rapamycin in *C. elegans*, three concentrations of the free drug (2, 6, and 15 mg/mL) were tested (Figure 5). As expected, Orlistat, used as a positive control, significantly reduced fat accumulation in the worms. In contrast, neither free rapamycin nor its encapsulated form in albumin nanoparticles altered fat levels compared to untreated controls. This seemingly unexpected result may be explained by the relatively low dose of rapamycin used, approximately three times lower than the minimum concentration (50 μM) previously reported to significantly reduce fat accumulation in worms under standard culture conditions.^65^

**Figure 5:**
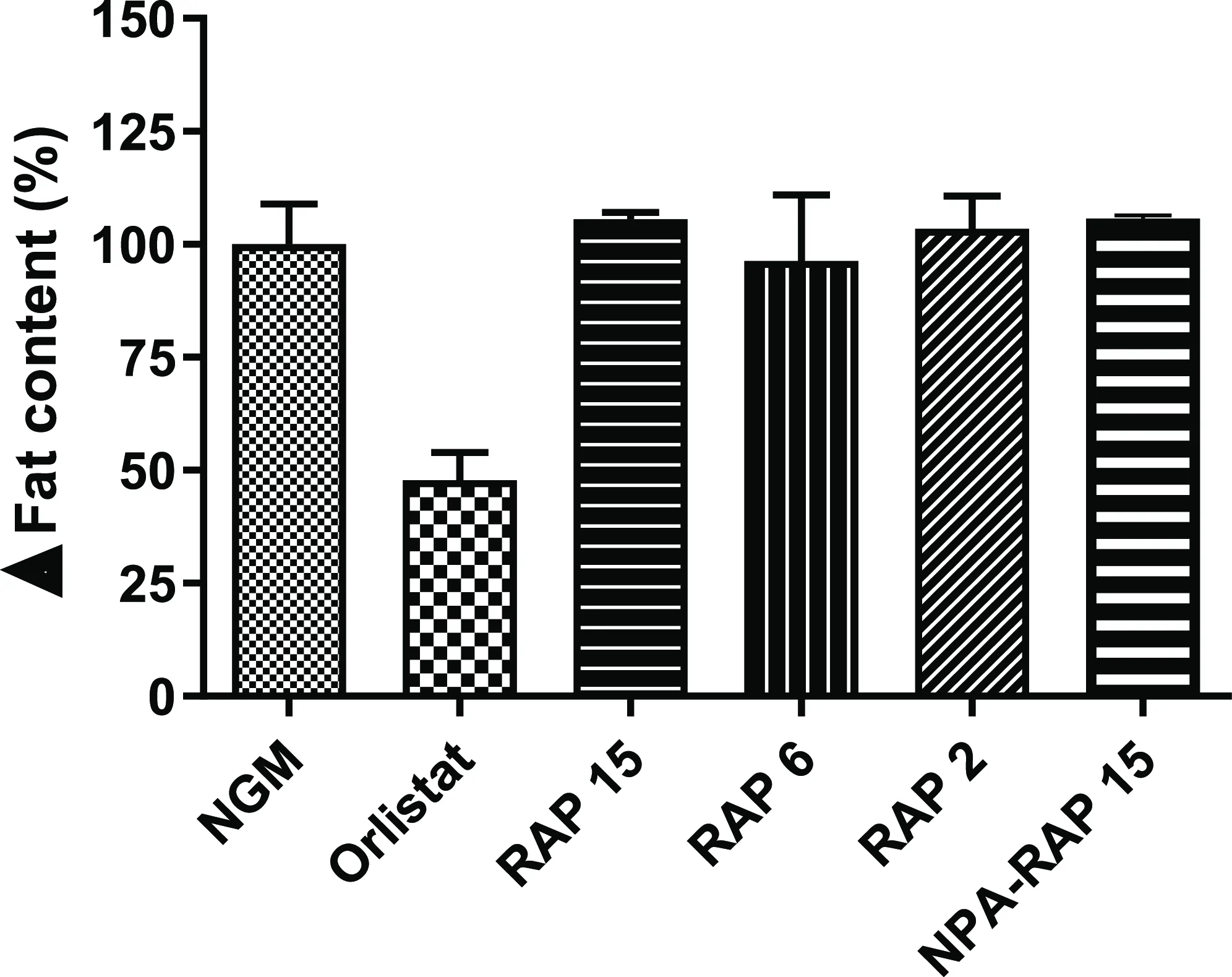
Effect of rapamycin on fat content in *C. elegans*. NGM: nematode growth medium; Orlistat (positive control); RAP (2, 6, and 15 mg/mL): free rapamycin; NPA-RAP15: albumin nanoparticles loaded with rapamycin. Results represent mean ± SEM from four replicates of 100 worms each (*n* = 400). Statistical analysis was performed using one-way ANOVA.

#### 3.3.2 Effect of Rapamycin-Loaded Nanoparticles on Lifespan

In addition to its metabolic effects, the impact of rapamycin on *C. elegans* longevity was evaluated (Figure 6 and Table 3). Worms maintained on standard NGM agar exhibited complete mortality by day 17, consistent with previous studies reporting a typical lifespan of 2–3 weeks.^63,70^ This is in line with the meta-analysis by Urban et al. (2021), which, based on over 200 studies, estimated the average lifespan of *C. elegans* at 17.7 days (95% CI: 17.33–18.06).^71^ Treatment with free rapamycin, compared with the control, extended the mean lifespan by approximately 27% and the maximum lifespan to about 23 days, in agreement with earlier findings.^63,72^ Notably, the nanoparticle-encapsulated formulation (NPA-RAP15) further increased mean lifespan by roughly 70% and the maximum lifespan to around 26 days, suggesting that nanoparticle-mediated delivery enhances the bioavailability and therapeutic efficacy of rapamycin.

**Figure 6:**
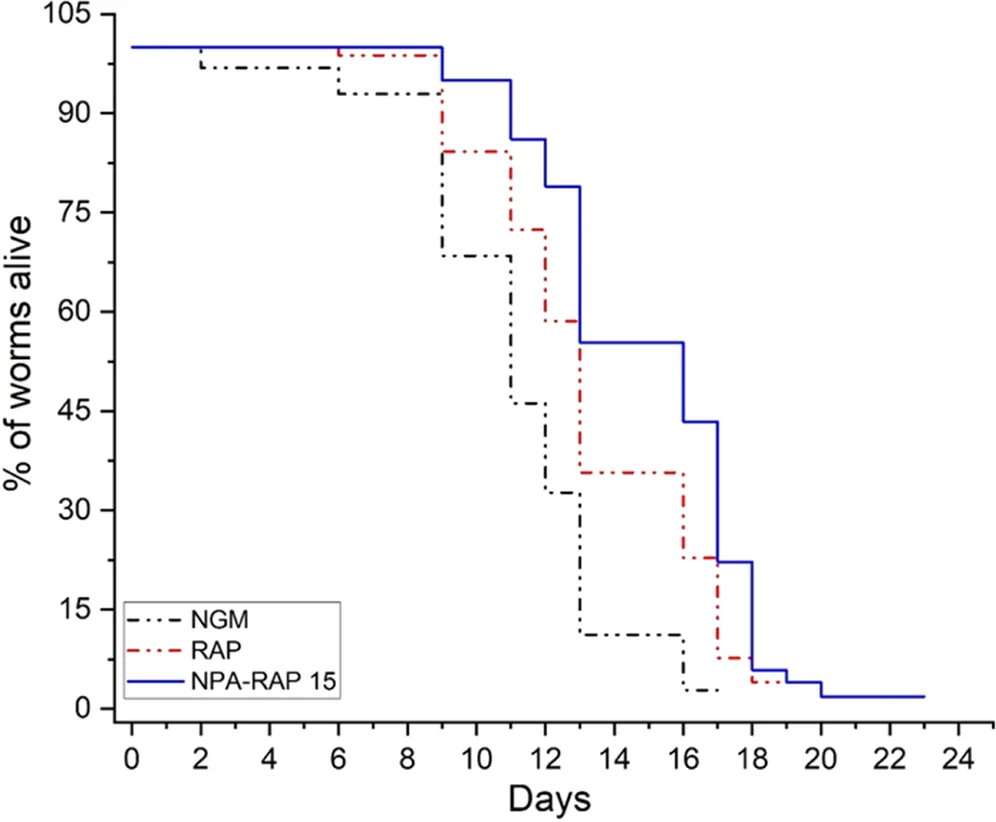
Survival curves of *C. elegans* (N2 strain) following treatment with NPA, free rapamycin, and rapamycin-loaded nanoparticles (NPA-RAP15). Worms were maintained on NGM agar, and survival was monitored daily. Results are expressed as mean survival ± SEM *n* = 100 worms per group.

**Table 3:** Effect of Oral Administration of Rapamycin (Free or Nanoencapsulated in Albumin Nanoparticles) on the Lifespan of *C. elegans* ^a^

	mean (days)	median (days)	maximum (days)
NGM	15 ± 2.0	15	17
RAP	19 ± 2.7*	19	23
NPA-RAP15	26 ± 0.6**	26	26

a *p < 0.05 and **p < 0.01compared to Control.

## 4 Conclusion

Rapamycin-loaded albumin nanoparticles were successfully developed with optimal physicochemical properties for intravenous delivery, including sizes within the tumor-targeting range (150–200 nm), low polydispersity (<0.2), and stable negative zeta potential (around—30 mV). PEGylation and surface charge contributed to excellent short-term stability. EE exceeded 70%, with sustained biphasic drug release (40% over 24 h) modeled effectively by the Weibull equation. In vitro, NPA-RAP15 showed cytotoxicity in MCF-7 breast cancer cells comparable to free rapamycin, with lower IC_50_ values, suggesting enhanced bioactivity. In *C. elegans*, neither free nor encapsulated rapamycin reduced fat content, likely due to low dosing. However, NPA-RAP15 markedly prolonged lifespan (∼70% increase in mean lifespan), outperforming free rapamycin and indicating improved therapeutic efficacy through nanoparticle delivery. All of these results indicate that the nanoparticle platform effectively delivers rapamycin while exhibiting no apparent carrier-related toxicity, supporting its potential translation to in vivo cancer models for further therapeutic evaluation.
